# Network analysis of comorbid mental health disorders among people with HIV in the All of Us Research Program

**DOI:** 10.21203/rs.3.rs-7473093/v1

**Published:** 2025-10-20

**Authors:** Victoria L. Money, Shan Qiao, Muyang Wu, Xiaoming Li

**Affiliations:** University of South Carolina

**Keywords:** HIV, comorbidity, psychometric network analysis, mental health disorders, EHR, ICD-10

## Abstract

**Background:**

People living with HIV experience high rates of mental health disorders, but the comorbidity patterns of these conditions remain poorly understood. Identifying how disorders cluster and which diagnoses are most central may guide more effective screening and integrated treatment strategies.

**Methods:**

This cross-sectional study used electronic health records and survey data from the National Institutes of Health All of Us Research Program (Release 8; May 2018–October 2023). Of 6,664 participants with confirmed HIV, 5,868 had electronic health record data; 3,078 with at least two encounters bearing mental health disorder diagnoses were included in the analysis. Diagnoses were identified using ICD-10 codes. Comorbidity networks were estimated with a psychometric network analysis based on conditional log-odds associations. Network centrality was evaluated using strength and expected influence measures, and clusters were identified with the Louvain algorithm. Sensitivity analyses included relaxing the diagnostic threshold to one recorded encounter and assessing robustness through nonparametric bootstrapping.

**Results:**

Participants were predominantly aged 50 years or older (69.2%), Black or African American (47.7%), non-Hispanic (77.5%), and male (64.4%); 91.2% had health insurance. The most common disorders were tobacco-related conditions (49%), major depressive disorder single episode (45%), other anxiety disorders (45%), recurrent depression (28%), adjustment and stressor-related disorders (25%), and substance-related conditions including alcohol (21%), cocaine (20%), and other psychoactive substances (19%). Two main clusters were identified: affective and anxiety-related disorders, and substance use and psychotic disorders. The most central diagnoses in the network were multiple psychoactive substance use, cannabis-related disorders, personality disorders, cocaine-related disorders, and recurrent depression. Sensitivity analyses supported the stability of the network structure and centrality rankings.

**Conclusions:**

Mental health disorders among people with HIV organize into two primary clusters centered on affective/anxiety and substance-related conditions. A small subset of highly central disorders, particularly recurrent depression and substance use conditions, appear to drive broader comorbidity patterns. Interventions targeting these central conditions may offer substantial benefit by disrupting interconnected clusters of psychiatric morbidity in this medically and socially vulnerable population.

## BACKGROUND

Owing to advances in both medication and public health approaches for HIV treatment, HIV has transitioned to a chronic disease requiring long-term care.^1–4^ As such, researchers and international organizations alike have identified critical and nuanced factors for HIV research and care.^5–7^ These include addressing the high prevalence of mental health disorders (MHD) and their unique comorbidity profiles.^4,8^ At the global level, 28–62% of PWH have at least one MHD, and in North America, approximately 55% of PWH have at least one MHD.^9,10^ This is especially concerning given that 21% of PWH in the United States have reported not receiving the mental healthcare they require.^11^

In general, about 50% of PWH have co-occurring MHDs, which can significantly impact their health outcomes and the effectiveness of HIV treatment plans.^10,12^ Within North America, PWH are more likely to have a MHD than their peers, with 24% exhibiting MHD comorbidities.^10^ MHD comorbidity refers to two or more mental health diagnoses occurring together. While each MHD may present its own set of challenges with specific treatment plans, the presence of one comorbidity increases the risk of developing others and should result in an integrated treatment approach that considers all conditions together.^13,14^ MHD comorbidities often lead to the need for multiple medications, which may have increased risks for physical, mental, and cognitive health.^15^ This emphasizes the critical need to combine mental health services with HIV-focused care, highlighting the pressing necessity to develop evidence-based and practical knowledge for screening, early detection, and initial clinical intervention while reducing potential risks.^9,16^

When assessing the prevalence of MHD comorbidities within the context of HIV, individual MHDs are often identified as key factors in subsequent treatment plans (e.g., care retention).^10,12,17,18^ Although this method provides pivotal insights into the most prominent MHDs experienced by PWH, it does not consider how different types of MHDs may be mutually reinforcing. If specific MHDs occur together, it may be insufficient to target them individually.^19–21^ To identify MHD comorbidities, common approaches include latent class analysis,^22–24^ principal class analysis,^25,26^ or regression analyses.^27^ Although these approaches offer key insights into the possible underlying factors and connections between MHDs, they do not establish whether specific MHDs are more prone to co-occur in relation to others. Psychometric network analysis (PNA), however, allows for comprehensive mapping of how MHD comorbidities are embedded within an interdependent system.^28–30^ This method provides a powerful approach for identifying which MHDs are more likely to co-occur. Thus, by studying how different MHDs are connected using PNA, we can better understand the links between them. This is especially important for those who are medically vulnerable and face social and/or legal challenges to support and care. For example, racial and ethnic minorities with HIV often have more difficulty obtaining diagnoses, treatment, and long-term care for both HIV and mental health issues.^31–35^

The present study leverages the All of Us dataset, which is uniquely suited for addressing these gaps. Designed to improve health equity, All of Us intentionally oversampled populations historically underrepresented in biomedical research, such as racial and ethnic minorities. This enables a more precise and inclusive analysis of comorbidity patterns within subgroups, which have often been overlooked in HIV and mental health research. Therefore, the current study aims to conduct a psychometric network analysis of MHDs among PWH based on electronic health records (EHR) and survey data extracted from the All of Us program. Specifically, we will (1) examine and visualize MHD comorbidity networks among PWH and (2) compare the centrality of each node (i.e., the MHD condition with the highest connectivity) within the network.

## METHOD

### Data source

We utilized data from the All of Us Research Program, a large, diverse, nationwide biomedical research initiative led by the National Institutes of Health (NIH) since 2018. The All of Us Program adopted the Observational Medical Outcomes Partnership (OMOP) Common Data Model (CDM) to harmonize data collected from over 710 institutional sites. More than 861,000 participants have contributed data to the OMOP CDM. Among them, over 746,000 participants completed all core enrollment steps. Before data are made available to researchers, all personal identifiable information (PII) is removed.^36,37^ The available EHR and survey data were harmonized and standardized through the OMOP CDM, ensuring consistency across data types and sources.^38^ These data are based on Release 8, including approximately 633,000 participants enrolled between May 6, 2018, and October 1, 2023.^39^ This cross-sectional analysis follows the STROBE reporting guidelines for observational settings.

### Included participants

The HIV cohort was identified using a validated computational phenotype algorithm developed as part of the All of Us Research Program.^36^ This algorithm integrated data from multiple sources, including diagnosis codes, drug exposures, laboratory measurements, and survey responses, to reliably classify individuals as having confirmed HIV infection. Specifically, participants were included if they met any of the following criteria: 1) presence of HIV-related diagnosis codes, 2) use of antiretroviral medications, 3) HIV-related laboratory results (e.g., viral load or CD4 counts), or 4) self-reported HIV status from surveys.

Mental health disorders were identified using ICD-10-CM codes (Supplementary Table 1). Both primary codes and their associated subcodes were included to comprehensively capture the full spectrum of mental health diagnoses. To ensure diagnostic validity, participants were considered to have a mental health condition if the relevant diagnostic code appeared on at least two separate dates during the study period. This approach aligns with established EHR-based phenotype definitions and enhances the accuracy of disorder classification.^40–42^

**Figure 1:**
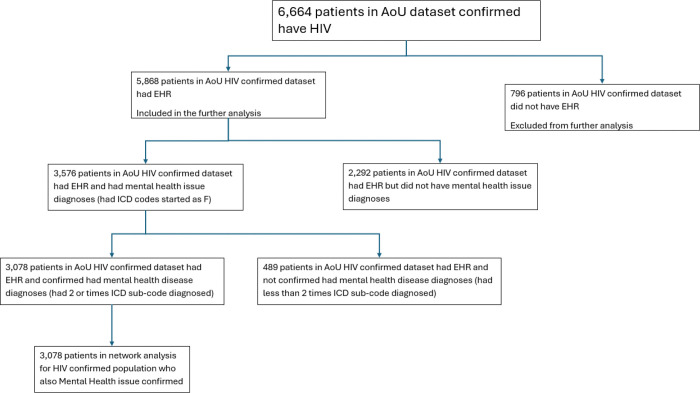
Flowchart of HIV-confirmed patient inclusion for mental health network analysis in the All of Us (AoU) dataset.

In total, 6,664 participants with confirmed HIV infection were identified in the All of Us dataset. Of these patients, 5,868 had EHR and were included in further analysis. Among those with EHR, 2,292 without any mental health codes and 489 with only one relevant code. The remaining 3,078 participants were confirmed to have mental health disorders and were included in the final psychometric network analysis. All participants were informed of data collection and provided consent, which was organized, collected, and is continuously monitored by the All of Us Research Program. Additionally, this study was approved by the institutional review board at the University of South Carolina (Pro00124044). Figure 1 shows the flowchart used to identify relevant participants for this study:

### Demographic variables

This data analysis also incorporated a range of demographic background variables, including age (e.g., 18–29, 30–39), sex assigned at birth, race, ethnicity, marital status, sexual orientation, educational attainment, annual household income, health insurance status, employment, and disability. These variables were obtained from both the EHR and participant-provided survey responses within the All of Us Research Program database.

### Analytic Strategy

All analyses were conducted using R software within the secure NIH Researcher Workbench, a cloud-based environment that allows approved users to access and analyze centralized, de-identified data.

### Descriptive Statistics

All demographic and socioeconomic variables were categorized into groups and summarized as frequencies and percentages. Additionally, we assessed the frequency of each mental health disorder ICD-code within this population.

### Psychometric network analysis

A psychometric network analysis (PNA) was conducted using the R *IsingFit* package to explore interdependent patterns of MHD among PWH.^43,44^ The ICD subcodes were mapped to their corresponding ICD parent codes, which were recorded as (Appendix 1) as binary indicators (0 = *absent*, 1 = *present*). This binarized matrix of ICD parent codes formed the basis for constructing the comorbidity network. PNA helps uncover the structure of disease co-occurrence by evaluating the conditional dependencies between diseases.^29,45–47^ Nodes in the network represent distinct conditions (ICD parent codes) and edges indicate the log odds of two nodes occurring together, controlling for all other co-occurrences in the network. The structure and likelihood of these connections provide insights into which conditions commonly co-occur and which conditions are salient within the network.

After identifying the strongest pairwise conditional associations, we measured each node’s strength and expected influence centrality score. The strength centrality score refers to where a node is positioned in relation to all other nodes; if node A has high strength centrality, that indicates it has a higher likelihood of co-occurring with many other nodes. The expected influence centrality score (EIC) refers to the summed edge weight connected to a node while retaining edge directionality.^48^ These metrics were computed to identify which nodes (i.e., diagnoses) are most likely to co-occur with all other nodes within the network. Although the input data were binary (i.e., indicating the presence or absence of a diagnosis), the edges in the network represent continuous-valued log-odds coefficients derived from regularized logistic regression.

Key visualizations were generated: (1) a psychometric network with community detection to highlight clusters of diseases that are more likely to co-occur together than with any other in the network; and (2) a bar charts for strength and EIC scores of the top five nodes simplified network showing only the top five strongest connections to emphasize the most critical comorbidity links. These visualizations aid in identifying central conditions (e.g., nodes with high connectivity or influence) and in understanding which comorbidities play pivotal roles within the health profiles of HIV-confirmed individuals with mental health diagnoses.

### Sensitivity analysis

To ensure the robustness of our findings and minimize the effects of potential misclassifications, we conducted a sensitivity analysis using nonparametric bootstrapping and report centrality correlation stability coefficients in the supplementary materials. In the primary analysis, individuals were classified as having a MHD only if the relevant ICD-10 diagnostic code was recorded on at least two separate dates. To evaluate whether our results were sensitive to this conservative criterion, we compared the results of the PNA for PWH with an MHD recorded on only one date instead of at least two.

## RESULTS

### Sample Characteristics

The majority of participants were 50 years or older, with 49.8% falling within the 50–64 age range and 19.4% being 65 or older. The racial distribution was primarily Black or African American (47.7%), followed by White (25.6%) and Asian/Other/Unknown (26.7%). Most participants identified as non-Hispanics (77.5%), while 18.2% identified as Hispanic or Latino. In terms of sex assigned at birth, 64.4% were male, 32.3% female, and 3.3% had unknown sex data. Regarding sexual orientation, 50.9% identified as straight, 44.3% as non-straight, and 4.8% declined to report or skipped the question. Among those surveyed, 91.1% indicated that they had completed high school or had attained a higher level of education. More than half of the participants (56.8%) indicated that their yearly income was under $25,000, whereas 23.2% reported earning more than that amount; income data were missing or unknown for 20.1% of participants. Most individuals were not employed for wages (76.4%). Health insurance coverage was high, with 91.2% of participants having some form of insurance. Finally, 21.2% of the cohort reported living with a disability.

### Mental Health Disorder Distribution

In this sample, nearly half (49%) had a documented diagnosis of tobacco-related disorders, making it the most prevalent mental health condition. This was closely followed by major depressive disorder (single episodes; 45%), and other anxiety disorders (45%). Recurrent major depressive disorder also affected over a quarter of the sample (28%), underscoring the burden of persistent depressive symptoms in PWH. Other highly prevalent MHDs included stress- and trauma-related disorders, such as adjustment disorders (25%), substance use disorders (including alcohol-related (21%), cocaine-related (20%), and other psychoactive substance use disorders (19%). These patterns resonate with the clustering of mood, anxiety, and substance use disorders among PWH, highlighting the need for integrated approaches to address the co-occurrence and mutual reinforcement of multiple psychiatric conditions in medically and socially vulnerable populations.

### Network Centrality and Comorbidity Structure

**Figure 2:**
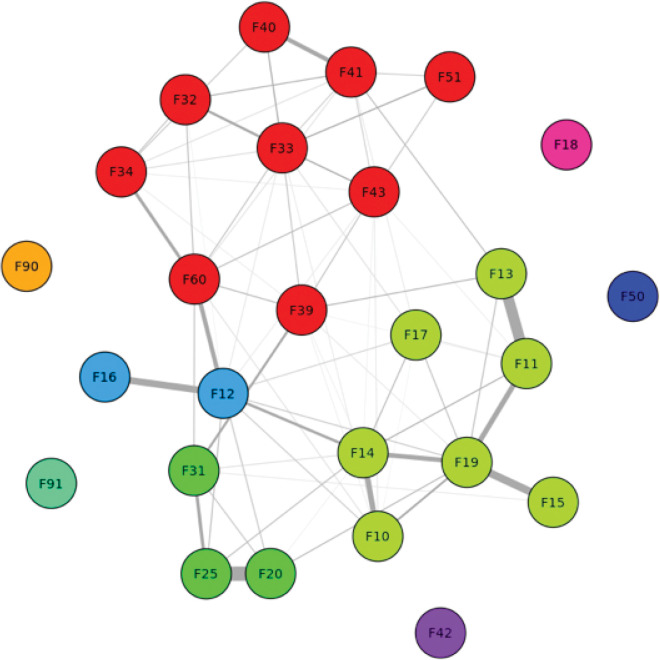
Community structure of mental health comorbidities among HIV-confirmed patients.

The overall structure of diagnostic co-occurrence is visualized in Fig. 2, where nodes represent ICD-10 parent codes, edges represent conditional associations between diagnoses, and edge thickness reflects the strength of these associations. Nodes are color-coded according to community membership, as determined by the Louvain clustering algorithm. A densely connected red cluster includes mood and anxiety-related disorders (e.g., F32, F33, F34, F39, F40, F41, F43, F50, F61), indicating a highly integrated internalizing disorder group. A prominent green cluster centers on substance use and psychotic disorders (e.g., F10, F11, F13, F14, F15, F17, F19, F20, F25, F31), reflecting a distinct pattern of comorbidity. Several nodes (e.g., F12, F16, F18, F42, F51, F90, F91) appear more peripheral, suggesting weaker or more isolated comorbidity relationships within the network.

**Figure 3:**
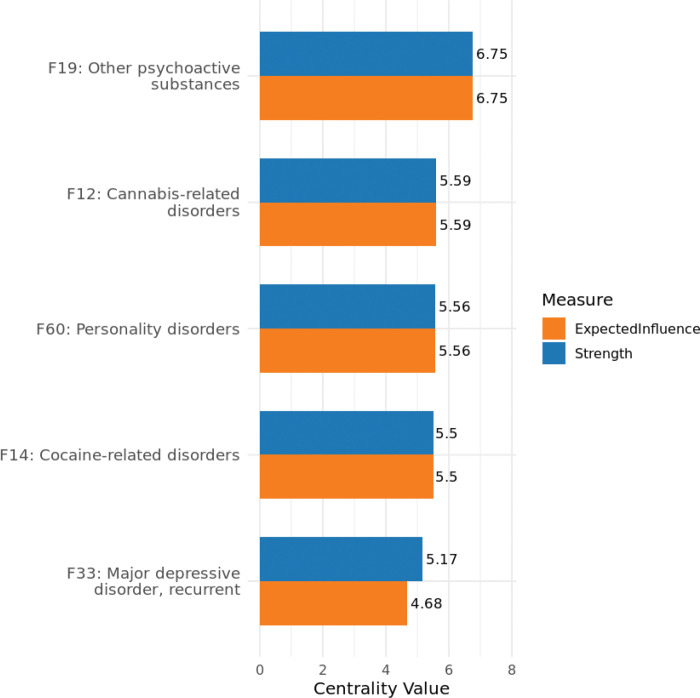
Top 5 ICD-10 parent codes ranked by strength centrality and expected influence.

Centrality values (strength and expected influence) for all ICD-10 codes are reported in Supplement 2. Among all diagnoses, five parent codes demonstrated the highest connectivity in the network: F19 (other psychoactive substance use disorders), F12 (cannabis-related disorders), F60 (personality disorders), F14 (cocaine-related disorders), and F33 (recurrent depressive disorder). As shown in Fig. 3, these diagnoses ranked highest in both strength and expected influence centrality, indicating that they co-occurred with a wide range of other MHDs. F19 and F12 consistently ranked highest, followed by F60 and F14, with F33 showing slightly lower expected influence relative to its strength. By contrast, several diagnoses, including F90 (attention-deficit hyperactivity disorder), F91 (conduct disorders), F50 (eating disorders), and F42 (obsessive-compulsive disorder), were positioned at the periphery of the network and exhibited limited or no direct associations with other MHDs, suggesting lower levels of diagnostic co-occurrence in this population.

### Sensitivity analysis

Sensitivity analysis demonstrated that the overall structure of the comorbidity network remained largely consistent when the inclusion criteria were expanded to encompass HIV-confirmed individuals with at least one ICD-10 code recorded at any time versus our study’s requirement of two or more diagnostic entries (See Supplement 3). The network continued to exhibit clearly defined clusters of MHDs and their associated comorbidities, with comparable patterns of connectivity and community organization across both models.

## DISCUSSION

This study presents a comprehensive psychometric network analysis of mental health diorders among people with HIV, using data from the All of Us Research Program. We identified a complex comorbidity structure marked by two major clusters of disorders: substance-related and affective/anxiety-related. Notably, the most central conditions in the network were F19 (other psychoactive substance use disorders), F12 (cannabis-related disorders), F60 (personality disorders), F14 (cocaine-related disorders), and F33 (recurrent depressive disorder). These diagnoses were not only among the most prevalent but also exhibited the highest strength and expected influence on centrality scores, indicating their frequent co-occurrence with a wide array of other mental health conditions.

The prominence of substance-related disorders, particularly F19, F12, and F14, suggests that substance use may function as both a central comorbidity and a potential driver of broader MHD clustering among PWH. This finding is particularly meaningful considering prior research suggesting that substance use may serve as a maladaptive coping strategy in response to chronic stress, stigma, trauma, or untreated depressive and anxiety symptoms.^49–51^ Substance use may exacerbate pre-existing neuropsychological conditions or contribute to the emergence of new ones, creating a reinforcing cycle of comorbidity. The centrality of personality disorders (F60) further supports the likelihood of enduring psychological and behavioral vulnerabilities that may underlie or compound these patterns of mental health risk. Importantly, these comorbidity patterns remained stable across sensitivity analyses, even when the diagnostic threshold for inclusion was relaxed. This suggests that the structure of MHD comorbidities among PWH is not an artifact of strict case definitions, but reflects meaningful, robust relationships within the population. The reliability of this network further underscores the need to prioritize central conditions, especially those that are modifiable or responsive to targeted interventions.

Clinically, these findings have clear implications. Integrated interventions that simultaneously address co-occurring depressive and substance-related disorders may have a cascading effect on the broader mental health burden experienced by PWH. Given their high centrality and influence, treating psychoactive substance, cannabis- and cocaine-related disorders (F19, F12, F14) along with depression and personality disorders may disrupt dense clusters of comorbidities and yield more sustainable outcomes than siloed treatment approaches. Given that over 40% of participants had depression and over 20% had cocaine-related disorders, targeting these high-centrality conditions could potentially reach nearly two-thirds of the mental health burden in this HIV cohort.

From a public health standpoint, the use of the All of Us dataset allowed for the inclusion of racially and socioeconomically diverse individuals, many of whom are underrepresented in biomedical research. The high prevalence of tobacco use, depression, and anxiety among these participants reinforces the importance of culturally responsive and equity-oriented care models that address both the psychosocial and structural determinants of health. Efforts to improve mental health outcomes among PWH must consider the broader cultural conditions, stigma, poverty, systemic racism, and healthcare inaccessibility, that shape comorbidity patterns.

In summary, the psychometric network analysis revealed that a small number of highly central MHDs, primarily substance use and affective disorders, serve as key hubs in the comorbidity network of PWH. Targeting these conditions in both clinical- and policy-level interventions may produce meaningful ripple effects across the mental health landscape of PWH.

Whereas this study provides clarity on what MHDs are most likely to co-occur among people with HIV, it uses cross-sectional data with intentional oversampling of individuals from underserved populations like racial and ethnic minorities. While this oversampling is a needed strength in medical research, findings from this study may not generalize to the broader US population. More importantly, establishing or mapping MHD comorbidity networks should not imply or assume causality based on demographic characteristics. Future studies should aim to uncover the underlying mechanisms that contribute to substance use and affective/mood disorders, such as economic and social disadvantage. This latter consideration and thus limitation of our study would not only contribute greatly to our understanding of MHD comorbidity but also the health-harming determinants of health that researchers can pinpoint as upstream factors that ultimately lead to cognitive and mental burden. Lastly, this comorbidity network serves as an exploratory analysis and would benefit from assessing whether specific clusters or combinations of MHDs differentially affect quality of life, psychological and physical well-being.

## CONCLUSION

In this psychometric network analysis of mental health disorders among people with HIV, a small subset of highly central conditions, particularly substance-related and affective disorders, emerged as key drivers of comorbidities. These findings suggest that interventions targeting central, co-occurring conditions may offer broader benefits by disrupting dense clusters of mental health burdens. Continued efforts to integrate culturally responsive, equity-oriented approaches will be critical for improving mental health outcomes in this medically and socially marginalized population.

## Supplementary Material

Supplementary Files

This is a list of supplementary files associated with this preprint. Click to download.

• HIVMHDSupplementalMaterialsMW.docx

## Figures and Tables

**Table 1:** Sample Population Characteristics

	Overall (N = 3087)
**Age Group**	
18–29	62 (2.0%)
30–39	361 (11.7%)
40–49	529 (17.1%)
50–64	1536 (49.8%)
65+	599 (19.4%)
**Race Group**	
Black or African American	1472 (47.7%)
White	790 (25.6%)
Asian/Other/Unknown	825 (26.7%)
Ethnicity Group	
Hispanic or Latino	562 (18.2%)
Not Hispanic or Latino	2391 (77.5%)
Unknown	134 (4.3%)
**Sex Assigned at Brith**	
Female	997 (32.3%)
Male	1988 (64.4%)
Unknown	102 (3.3%)
**Sex Orientation**	
Sexual Orientation: Non-straight	1368 (44.3%)
Sexual Orientation: Straight	1570 (50.9%)
Unknown	149 (4.8%)
**Education**	
High school degree or more	2813 (91.1%)
Less than a high school degree	130 (4.2%)
Unknown	144 (4.7%)
**Income**	
Greater than 25,000 US dollars	716 (23.2%)
Less than 25,000 US dollars	1752 (56.8%)
Unknown	619 (20.1%)
**Employment Status**	
Employed for wages/self-employed	728 (23.6%)
Not employed for wages	2359 (76.4%)
**Health Insurance**	
No	167 (5.4%)
Yes	2814 (91.2%)
Unknown	106 (3.4%)
**Disability**	
No	2432 (78.8%)
Yes	655 (21.2%)

**Table 2:** Mental Health Disorder Frequency

ICD Code	Mental Health Disorder	Count	Percent	Total
F17	Tobacco-related disorders	1512	49	3087
F32	Major depressive disorder, single episode	1402	45.4	3087
F41	Other anxiety disorders	1393	45.1	3087
F33	Major depressive disorder, recurrent	861	27.9	3087
F43	Reaction to severe stress and adjustment disorders	757	24.5	3087
F10	Alcohol-related disorders	658	21.3	3087
F14	Cocaine-related disorders	622	20.1	3087
F19	Other psychoactive substances	571	18.5	3087
F31	Bipolar disorder	511	16.6	3087
F11	Opioid-related disorders	448	14.5	3087
F51	Sleep disorders	293	9.5	3087
F15	Other stimulant-related disorders	292	9.5	3087
F12	Cannabis-related disorders	288	9.3	3087
F39	Unspecified mood [affective] disorder	280	9.1	3087
F20	Schizophrenia and other psychotic disorders	204	6.6	3087
F25	Schizoaffective disorders	158	5.1	3087
F34	Dysthymic disorder/Cyclothymic disorder/Other persistent mood [affective] disorders	148	4.8	3087
F60	Personality disorders	142	4.6	3087
F90	Attention-deficit hyperactivity disorder	136	4.4	3087
F40	Specified phobia/Anxiety disorders	77	2.5	3087
F13	Sedative-related disorders	68	2.2	3087
F50	Eating disorder	29	0.9	3087
F91	Conduct disorders	17	0.6	3087
F42	Obsessive-compulsive disorder	14	0.5	3087
F16	Hallucinogen-related disorders	13	0.4	3087
F18	Inhalant-related disorders	6	0.2	3087
